# Immobilized probe and glass surface chemistry as variables in microarray fabrication

**DOI:** 10.1186/1471-2164-5-53

**Published:** 2004-08-04

**Authors:** Martin J Hessner, Lisa Meyer, Jennifer Tackes, Sanaa Muheisen, Xujing Wang

**Affiliations:** 1 The Max McGee National Research Center for Juvenile Diabetes, Department of Pediatrics, The Medical College of Wisconsin and Children's Hospital of Wisconsin, 8701 Watertown Plank Road, Milwaukee, WI 53226, USA; 2 The Human and Molecular Genetics Center, The Medical College of Wisconsin, 8701 Watertown Plank Road, Milwaukee, WI 53226, USA

## Abstract

**Background:**

Global gene expression studies with microarrays can offer biological insights never before possible. However, the technology possesses many sources of technical variability that are an obstacle to obtaining high quality data sets. Since spotted microarrays offer design/content flexibility and potential cost savings over commercial systems, we have developed prehybridization quality control strategies for spotted cDNA and oligonucleotide arrays. These approaches utilize a third fluorescent dye (fluorescein) to monitor key fabrication variables, such as print/spot morphology, DNA retention, and background arising from probe redistributed during blocking. Here, our labeled cDNA array platform is used to study, 1) compression of array data using known input ratios of *Arabidopsis in vitro *transcripts and arrayed serial dilutions of homologous probes; 2) how curing time of in-house poly-L-lysine coated slides impacts probe retention capacity; and 3) the retention characteristics of 13 commercially available surfaces.

**Results:**

When array element fluorescein intensity drops below 5,000 RFU/pixel, gene expression measurements become increasingly compressed, thereby validating this value as a prehybridization quality control threshold. We observe that the DNA retention capacity of in-house poly-L-lysine slides decreases rapidly over time (~50% reduction between 3 and 12 weeks post-coating; p < 0.0002) and that there are considerable differences in retention characteristics among commercially available poly-L-lysine and amino silane-coated slides.

**Conclusions:**

High DNA retention rates are necessary for accurate gene expression measurements. Therefore, an understanding of the characteristics and optimization of protocols to an array surface are prerequisites to fabrication of high quality arrays.

## Background

The generation of reliable gene expression data with cDNA microarrays requires fabrication of quality arrays. This task encompasses the amplification of adequate amounts of concentrated PCR product for use as probe from the cDNA clone, followed by ordered arraying of the probes onto coated glass slides. The glass slide is a key variable in either spotted cDNA or oligonucleotide array fabrication since it must possess: 1) a uniform surface that yields spots of consistent shape and size, 2) low background fluorescence, and 3) high DNA retention capacity. Since the array is clearly a source of experimental variability, we have developed a novel three-color array approach where it is possible to directly visualize either cDNA or oligonucleotide arrays prior to hybridization [1-3]. For cDNA arrays, the probes are easily tagged with a third, Cy3/Cy5 compatible, fluorescent dye (fluorescein) during amplification. After purification of PCR products, which includes removal of unincorporated oligonucleotide primer, the detected fluorescein fluorescence represents deposited cDNA probe on the array. This three-color approach allows for assessment of slide fabrication independent of hybridization, thereby enabling 1) direct visualization of array/element morphology, 2) quantification of probe deposition and retention on the slide surface and 3) ultimately a means for array quality control prior to hybridization.

By labeling the array itself with a third color, we have observed that arrays fabricated together are not equivalent in terms of a number of measurable physical parameters, including the amount of DNA probe deposited and retained and the amount of background arising from probe solublized and re-deposited during post-processing. In prior studies, we observed that these pre-hybridization array-based variables play a direct and significant relationship in replicate consistency, and that microarray data quality can be improved through prehybridization slide selection based upon these quality parameters [1,2]. As a result of these studies, we identified putative slide acceptance criteria: array fluorescein mean element intensity >5000 RFU/pixel, coefficient of variation (CV) in intensity <10%, mean signal to noise score (signal/signal + noise; S/S+N) >0.85, and CV in spot size <20%. In this report, using known input ratios of *in vitro *transcript we experimentally correlate the quantity of support bound probe to measured expression ratios, in order to validate our quality control threshold for array acceptance. We then utilize our three-color array platform to evaluate the characteristics of in-house prepared poly-L-lysine coated slides and 13 additional commercially available coating surfaces, in terms of background auto-fluorescence, spot morphology, and DNA retention.

## Results and Discussion

### The relationship between support bound probe and measured ratio reliability

It has been assumed that the amount of cDNA probe deposited and retained on the array surface would have a nominal effect on observed differential expression ratios due to the competitive nature of two channel fluorescent hybridizations [4]; however this assumption has been shown to be false [1,5]. Yue et al., using unlabeled *Saccharomyces cerevisiae *probes and complementary Cy5 and Cy3 labeled cDNA targets derived from *in vitro *transcripts, indirectly demonstrated this by printing yeast probes at increasingly dilute concentrations (<50 ng/ul) and observed elimination of the measured dynamic range to where input transcript ratios of 30:1 or 1:30 were both detected as output ratios close to 1:1, illustrating that limiting bound probe results in an underestimation or failure to detect differential gene expression [5].

**Figure 1:**
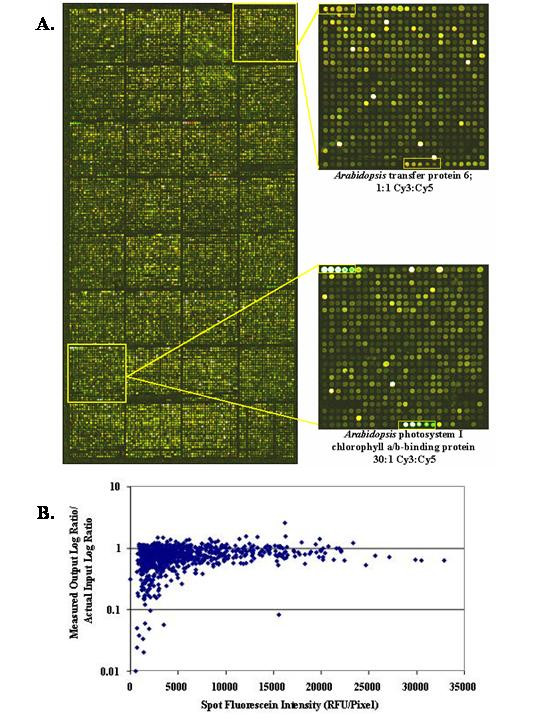
Evaluation of measured output ratio of spiked *Arabidopsis in vitro *transcript at known input ratios. A. Total thymus RNA extracted from the DR+/+ and DR*lyp/lyp *[6] BioBreeding rats spiked with known input ratios of *Arabidopsis *gene *in vitro *transcript and hybridized to 18,000 probe rat cDNA arrays possessing serially diluted fluorescein-labeled *Arabidopsis *probes. B. Evaluation of data compression as a function of support-bound probe. On the x-axis is plotted the average pixel fluorescein intensity per spot plotted against the *Arabidopsis *transcript measured output log ratio/actual input log ratio. As spot intensities fall below 5000 RFU/pixel, ratio measurements become increasingly compressed.

To expand upon these observations and place them in context with the quality control standards of our three-color array platform, we conducted similar experiments using *Arabidopsis *probes and transcripts. Total thymus RNA extracted from the DR+/+ and DR*lyp/lyp *[6] BioBreeding rats was directly labeled through reverse transcription reactions possessing cyanine dyes; these labeling reactions were spiked with known input ratios (30:1, 10:1, 1:1, and 1:0) of *Arabidopsis *gene *in vitro *transcript and hybridized to 18,000 probe rat cDNA arrays possessing serially diluted fluorescein-labeled *Arabidopsis *probes (cellulose synthase, chlorophyll a/b binding protein, and ribulose-1,5-bisphosphate and triosphosphate isomerase, 1:2 dilution series printed at 200 ng/ul to 6.25 ng/ul). This approach allowed comparison of known RNA input ratio to measured output ratio, enabling a direct and quantitative measure of the relationship between the amount of support-bound probe and ratio data compression (Figure 1A and 1B). Using Matarray [7,8], spots possessing low hybridized image quality (*q*_com_) were filtered; these spots were either saturated or possessed high background. Spots with low hybridization intensities, which would normally be flagged by Matarray, were intentionally retained to study ratio compression due to low amounts of support-bound probe. After filtering, 896/1536 data points from 16 different arrays were available for analysis. Plotted on the y-axis of Figure 1b, is the measured *Arabidopsis in vitro *transcript output log ratio divided by the log ratio of transcript actually introduced into the Cy3 and Cy5 labeling reactions. In this analysis, a perfect measurement is represented by a value of "1". On the x-axis, is plotted the spot fluorescein intensity. When the spot fluorescein intensity falls below 5000 RFU/pixel, the data variability and data compression (underestimation of differential gene expression) dramatically increase. These results recapitulate our previous observations where replicate consistency was found to decrease when the array average spot fluorescein intensity dropped below 5000 RFU/pixel, whereas arrays possessing average fluorescein intensities above 5000 RFU/pixel were found to generate equally good data. These results further demonstrate that use of measurable array characteristics are effective quality markers for printed arrays (judged by their effect on the hybridization data) and serve to validate our array intensity quality control threshold of >5000 RFU/pixel.

### Impact of poly-L-lysine cure-time on DNA retention capacity

Clearly, the amount of immobilized probe on the coated glass surface is a critical array fabrication variable, therefore factors that affect the amount of retention characteristics, such as surface chemistry, probe concentration, spotting buffer, spotting conditions, cross-linking and blocking conditions are important to understand. Protocols for coating glass microscope slides with poly-L-lysine are readily available on-line and reasonably simple to perform (for example: ; ; ). Although most available protocols are quite similar, some recommend the curing of slides for two weeks prior to spotting, while others state that coated slides are not stable for extended periods of time and recommend not printing onto slides that are greater than 4 months old. To investigate slide coating age as a potential variable in retention capacity, we fabricated more than 1,000 rat cDNA arrays (18,000 element/slide) using in-house poly-L-lysine coated slides ranging in age from 3 to 12 weeks. These slides were coated in 26 independent sessions and utilized over 12 different print runs. After printing all arrays were post-processed [9] and imaged under standardized conditions as previously described [1,2].

**Figure 2:**
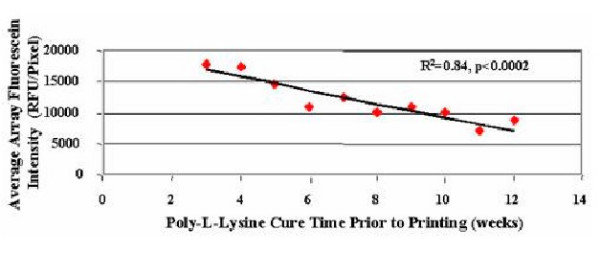
Relationship between DNA retention and poly-L-lysine cure time. Retention capacity is lost as the poly-L-lysine cure time increases (R^2 ^= 0.84; p < 0.0002). Analysis includes 999 arrays printed over 12 different print runs. Each print run consisted of 100 arrays, printed onto poly-L-lysine slides from 2 or more coating lots.

A significant loss of DNA retention capacity is observed (Figure 2) when the average array spot fluorescein intensity is plotted against the coating age at the time of printing (R^2 ^= 0.84; p < 0.0002). An average array fluorescein intensity of >15,000 RFU/pixel was observed when printing on slides 4 weeks old or less, however a nearly 50% reduction in retention capacity is observed when printing on poly-L-lysine coating greater than 10 weeks old. We speculate that the poly-L-lysine may become oxidized thereby losing its positive charge and ability to initially electrostatically interact with the negatively charged DNA. Irregardless of the type of degradation occurring to the surface coating over time, these results indicate that, at least for in house fabricated poly-L-lysine coated slides, shelf-life is a significant variable in the fabrication of quality arrays capable of yielding reliable gene expression measurements.

### Investigation of probe retention characteristics of commercially available coated slides

Given the potential time-dependent variability of in-house prepared poly-L-lysine coated slides, we investigated the retention characteristics of commercially available coated slides. Our objective was to identify a surface with consistently higher retention characteristics than our "fresh" in-house slides without having to change the spotting buffer (1.5 M betaine/5% DMSO) or the nonaqueous post-processing protocol [1,2,9], since these methods were previously found to yield high quality results on poly-L-lysine coated slides prepared in-house. We obtained examples of 13 different vendor-supplied slides for evaluation that possessed either poly-L-lysine, aminosilane, or undisclosed surface chemistries. Prior to printing, background auto-fluorescence in the fluorescein, Cy3, and Cy5 channels was evaluated. Fluorescein auto-fluorescence was observed on all poly-L-lysine slides except for those produced in-house, as well as 6 of the aminosilane slides (Asper Biotech, Corning, Erie Scientific, Genetix, Telechem), and the proprietary surface from Full Moon Biosciences. Cy3 auto-fluorescence was observed on all 3 commercial poly-L-lysine slides but not those prepared in-house, however none was observed on any of the aminosilane slides. Insignificant background in the Cy5 channel was only observed on 2 commercial poly-L-lysine slides (Electron Microscopy Sciences, Polysciences Inc.).

**Figure 3:**
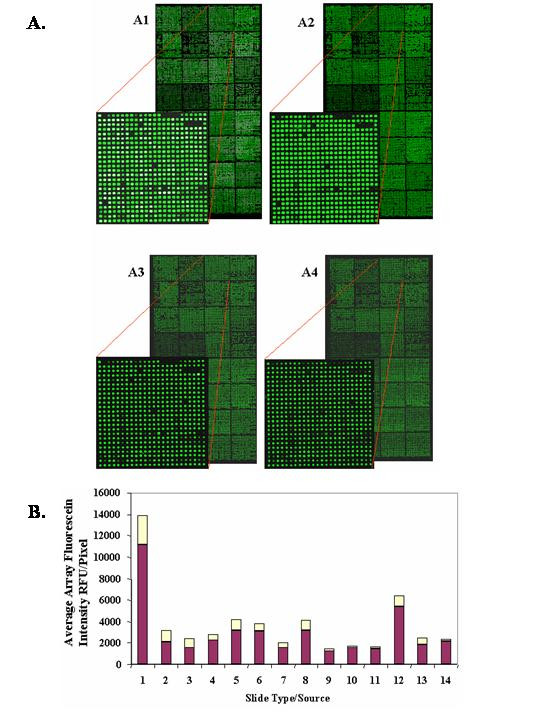
A: Fluorescein images of 18,000 element rat cDNA arrays on in-house poly-L-lysine coated slide after printing (A1) and array after non-aqueous post-processing (A2). Fluorescein images of simultaneously 18,000 element rat cDNA arrays on Full Moon Biosystems coated slide (undisclosed chemistry) after printing (A3) and array after non-aqueous post-processing (A4). Note differences in amount of DNA deposited and retained. (White spots are saturated). B: Comparison of retention capacity of 14 different coating surfaces using human 9,600 probe cDNA arrays. Tabulated measurements are based upon 5 replicates slides (~48,000 elements) for each slide type evenly distributed over the arrayer deck (ie 5 slides of a given type did not occupy 5 adjacent positions on the arrayer deck). Slides 1–4 are poly-L-lysine: MCW in-house, Electron Microscopy Sciences, Polysciences, and Cel Associates, respectively. Slides 5–13 are aminated: Asper Biotech, Apogent, Bioslide, Erie Scientific, Genetix, Corning Ultra GAPS, Corning GAPS II, Sigma, and Telechem Super Amine, respectively. Slide 14 is an undisclosed chemistry offered by Full Moon Biosystems. The graph represents the average spot fluorescein intensity RFU/pixel (burgundy) +/- standard deviation (yellow).

To study retention characteristics, a single 9600 element human cDNA array was spotted onto each slide in 1.5 M betaine/3%DMSO. The in-house poly-L-lysine slides were less than 6 weeks old and all vendor-supplied slides were unpacked from any special packaging immediately before printing. Five replicate arrays for each slide type were generated. The five replicates were evenly distributed over the arrayer deck (capacity 100 slides) by arranging the slides into 5 groups of 18 to account for any variance introduced through print rank order (ie first versus last), since we previously identified this as a variable that influences array average array fluorescein intensity [1]. Fluorescein images were again obtained, under strict standardized conditions, immediately after printing and again after post-processing to measure DNA deposited and retained (Figure 3A; Table 1) [1,2]. The average amount of DNA deposited per element varied considerably among slides of different sources (n = 5 per source) ranging from a low of 2,300 +/-300 RFU/pixel to a high of 20,700 +/-3,300 RFU/pixel. Among the slides evaluated, the poly-L-lysine coated slides yielded larger spot sizes, perhaps due to a lower hydrophobicity than the aminated surfaces. The average DNA retained per element after blocking/post-processing ranged from a low of 1,400 +/-200 RFU/pixel to a high of 11,100+/-2,700 RFU/pixel on poly-L-lysine slides prepared in-house (Figure 3B). These results, combined with our previous observations [1,2], indicate that DNA concentration, choice of printing buffer, slide position on the arrayer deck and slide surface chemistry all influence the amount of DNA deposited and ultimately retained, which can be effectively monitored by our three-color approach. We have previously shown that the amount of probe solublized and redistributed over the slide during post-processing is an important quality control parameter [2]. Eight of the surfaces tested generated fluorescein signal to noise values (signal/signal + noise; S/S+N) ≥ 0.90 after post-processing, a value that we have previously shown to sufficient to generate high replicate reproducibility [2]. However, the majority of vendor-supplied coated slides, did not meet or exceed our established average array element intensity value of 5,000 fluorescein RFU/pixel under the printing and post-processing methods optimized for our in-house coated slides. These results point towards the possibility that array fabrication should be optimized to the specific surface selected for use and that even the same surface chemistry from different sources may perform differently.

**Table 1:** Retention Studies on Commercial Coated Slide Surfaces

Vendor	Chemistry	RFU/Pixel deposited × 10^3^	RFU/pixel retained × 10^3^	Percent Retention	Spot Diameter	Processed S/(S+N)
MCW in-house	Poly-L-lysine	19.0+/-7.7	11.1+/-2.7	58.4%	114+/-9	0.90+/-0.00
Electron Microscopy Sciences	Poly-L-lysine	15.3+/-4.9	2.12+/-1.1	13.9%	100+/-28	0.85+/-0.04
Polysciences	Poly-L-lysine	11.5+/-6.3	1.3+/-1.0	11.3%	125+/-6	0.86+/-0.06
Cel Associates	Poly-L-lysine	5.7+/-3.6	2.3+/-0.5	40.4%	100+/-35	0.79+/-0.09
Asper Biotech	Aminated	6.8+/-2.4	3.2+/-1.1	47.1%	98+/-22	0.93+/-0.05
Apogent Ezrays	Aminated	6.0+/-2.9	2.8+/-0.3	46.7%	109+/-20	0.92+/-0.02
Bioslide	Aminated	3.3+/-0.9	1.5+/-0.5	45.5%	93+/-7	0.87+/-0.02
Erie Scientific	Aminated	11.5+/-4.1	3.2+/-0.9	27.8%	81+/-15	0.90+/-0.01
Genetix	Aminated	4.1+/-1.2	1.2+/-0.2	29.3%	94+/-23	0.86+/-0.02
Corning Ultra GAPS	Aminated	2.3+/-0.3	1.5+/-0.2	65.2%	103+/-13	0.92+/-0.01
Corning GAPS II	Aminated	3.9+/-0.9	1.4+/-0.2	35.9%	117+/-18	0.92+/-0.01
Sigma	Aminated	20.7+/-3.3	5.4+/-1.0	26.1%	93+/-8	0.79+/-0.01
Telechem Super Amine	Aminated	6.7+/-2.1	1.7+/-0.5	25.4%	107+/-19	0.80+/-0.0
Full Moon Biosystems	Proprietary	6.4+/-1.4	2.1+/-0.2	32.8%	78+/-7	0.90+/-0.1

It has been reported that the amount of UV irradiation may be an important array fabrication variable since the amount of hybridization signal from spotted 70-mer oligonucleotides has been found to be dependent on the amount of cross-linking [10]. In this previous report, different optimal cross-linking intensities for attachment of spotted 70-mer oligonucleotides were observed for different slide coating chemistries (poly-L-lysine, aldehyde, aminosilane, epoxide) [10]; furthermore, different cross-linking optima for probe attachment were also observed for slides with the same or similar slide chemistry from different vendors. This variable was not explored in our evaluation of vendor-supplied surfaces and may account for some of the performance differences observed. The report by Wang et al., [10] prompted us to revisit this parameter for our in-house slides and we have observed approximately 20% better DNA retention by increasing the UV cross-linking energy from 60 mJ/cm2 to 200 mJ/cm2 independent of coated slide lot.

Fabrication of high quality spotted arrays is a daunting task possessing a high number of variables. The vendor supplied slides tested here were done so under conditions that have been optimized for our in-house prepared poly-L-lysine coated slides, although our optimized protocol is not drastically different than those used by other laboratories nor drastically different from any of the vendor provided protocols. Our observations, as well as the observations of others, suggest that optimization of ones protocol to a surface chemistry is an essential first step to generating reliable global gene expression measurements using in-house spotted microarrays.

## Methods

A sequence-verified human library (Research Genetics, Huntsville, AL), consisting of 41,472 clones or a 36,000 clone rat cDNA library obtained from the University of Iowa was used as a source of probe DNA. Cultures were grown in 150 ul Terrific Broth (Sigma, St. Louis, MO) supplemented with 100 mg/ml ampicillin in 384 deep-well plates (Matrix Technologies, Hudson, NH) sealed with air pore tape sheets (Qiagen, Valencia, CA) and incubated with agitation for 14–16 hr. Clone inserts were amplified in duplicate in 384-well format from 0.5 ul bacterial culture or from 0.5 ul purified plasmid (controls only) using 0.26 μM of each vector primer (SK865 5'-fluorescein-GTC CGT ATG TTG TGT GGA A-3' and SK536: 5'-fluorescein-GCG AAA GGG GGA TGT GCT G-3' [5]) (Sigma-Genosys, The Woodlands, TX) in a 20 μl reaction consisting of 10 mM Tris-HCl pH8.3, 3.0 mM MgCl_2_, 50 mM KCl, 0.2 mM each dNTP (Amersham, Piscataway, NJ), 1 M betaine [11,12], and 0.50 U *Taq *polymerase (Roche, Indianapolis IN). Reactions were amplified with a touchdown thermal profile consisting of 94°C for 5 min; 20 cycles of 94°C for 1 min, 60°C for 1 min (minus 0.5° per cycle), 72°C for 1 min; and 15 cycles of 94°C for 5 min; 20 cycles 94°C for 1 min, 55°C for 1 min, 72°C for 1 min; terminated with a 7 min hold at 72° [13-15]. PCR reactions were analyzed for single products by 1% agarose gel electrophoresis. Products from replicate plates were pooled and then purified by size exclusion filtration using the Multiscreen 384 PCR filter plates (Millipore, Bedford, MA). Forty wells of each 384-well probe plate were quantified by the PicoGreen assay (Molecular Probes, Eugene, OR) according to the manufacturers instructions. After quantification, all plates were dried down, and reconstituted at 125 ng/μl in 3% DMSO/1.5 M betaine. It has been shown that betaine normalizes base pair stability differences, increases solution viscosity, reduces evaporation rates [11], and enhances probe binding to surfaces such as poly-L-lysine or aminosilane [1,9]. We have observed higher probe retention at much lower DNA concentrations (150–200 ng/ul) in the presence of betaine versus the typically required 4–500 ng/ul when using conventional printing solutions [2,3].

*Arabidopsis thaliana *PCR product and *in vitro *transcript were purchased from Stratagene (La Jolla, CA) as part of the SpotReport^®^-10 Array Validation System. *Arabidopsis thaliana *PCR product was cloned into the pCRII vector using the TA cloning kit (Invitrogen, Carlsbad CA) and fluorescein-labeled PCR products for photosystem I chlorophyll a/b-binding protein, RUBISCO activase, ribulose-1,5-biphosphate carboxylase/oxygenase, lipid transfer protein 6 lipid transfer protein 5, papain-type cysteine endopeptidase, root cap 1, and triosphophate isomerase were generated using vector-specific primers essentially as described above. Products were purified, quantified, and a 1:2 dilution series (200 ng/ul to 12.5 ng/ul) was prepared and printed in duplicate onto each array.

Poly-L-lysine coated slides were prepared in-house as previously described [16] on Corning (Corning, NY) pre-cleaned 75 × 25 mm glass micro slides. Nine different commercially available aminosilane coated slides (Apogent Discoveries, Waltham, MA; Asper Biotech, Redwood City, CA; Bioslide Technologies, Walnut, CA; Corning Inc, Corning NY; Erie Scientific, Portsmouth, NH; Genetix, St. James, NY; Sigma, St. Louis, MO; Telechem International Inc, Sunnyvale, CA) and 3 different commercially available poly-L-lysine coated slides (Cel-Associates, Pearland, TX; Electron Microscopy Sciences, Fort Washington, PA; Polysciences Inc., Warrington, PA) were obtained for evaluation. Additionally, slides coated with a proprietary chemistry (Full Moon Biosystems, Sunnyvale, CA) were obtained. Microarrays possessing a density of 9,600 human probes/slide were printed onto coated slides using a GeneMachines Omni Grid printer (San Carlos, CA) with 16 Telechem International SMP3 pins (Sunnyvale, CA) at 40% humidity and 22°C. To control pin contact force and duration, the instrument was set with the following Z motion parameters, velocity: 7 cm/sec, acceleration: 100 cm/sec^2^, deceleration: 100 cm/sec^2^. All slides were post-processed using the previously described non-aqueous protocol [9] using 60 mJ/cm^2 ^UV cross-linking energy. This protocol has yielded more favorable fluorescein post-blocking signal-to-noise values (signal/signal+noise; S/S+N) as compared to blocking in aqueous solutions[2]. Image files on all slides were collected prior to printing to establish background fluorescence (fluorescein, Cy3 and Cy5), after printing (fluorescein), and after blocking (fluorescein), with a ScanArray 5000 (GSI Lumonics, Billerica, MA). Array image files were analyzed with the Matarray software [7,8,17].

Isolation of mRNA, labeling, and hybridization were performed as described previously . Known input ratios of photosystem I chlorophyll a/b-binding protein (30:1); RUBISCO activase (10:1); ribulose-1,5-biphosphate carboxylase/oxygenase (5:1); lipid transfer protein 6 (1:1); 0.7 lipid transfer protein 5 (1:1); papain-type cysteine endopeptidase (1:5); root cap 1 (1:10); and triosphophate isomerase (1:30) were spiked into Cy3 and Cy5 RNA labeling reactions, respectively. After hybridization, arrays were scanned with a ScanArray 5000 (GSI Lumonics, Billerica, MA) and image files were obtained. Again, array image files were analyzed with the Matarray software [7,8,17].

## Authors' Contributions

MJH and XW conceived of the study, its design and coordination, and drafted the manuscript. LM, JT, and SM carried out the array fabrication and gene expression studies. XW performed the statistical analysis. All authors read and approved the final manuscript.
